# Site-specific phosphorylation of myosin binding protein-C coordinates thin and thick filament activation in cardiac muscle

**DOI:** 10.1073/pnas.1903033116

**Published:** 2019-07-15

**Authors:** Saraswathi Ponnam, Ivanka Sevrieva, Yin-Biao Sun, Malcolm Irving, Thomas Kampourakis

**Affiliations:** ^a^Randall Centre for Cell and Molecular Biophysics, King’s College London, SE1 1UL London, United Kingdom;; ^b^British Heart Foundation Centre of Research Excellence, King’s College London, SE1 1UL London, United Kingdom

**Keywords:** cardiac muscle regulation, myosin binding protein-C, phosphorylation

## Abstract

The heart’s response to varying demands of the body is regulated by signaling pathways that activate protein kinases which phosphorylate sarcomeric proteins. Although phosphorylation of cardiac myosin binding protein-C (cMyBP-C) has been recognized as a key regulator of myocardial contractility, little is known about its mechanism of action. Here, we used protein kinase A (PKA) and Cε (PKCε), as well as ribosomal S6 kinase II (RSK2), which have different specificities for cMyBP-C’s multiple phosphorylation sites, to show that individual sites are not independent, and that phosphorylation of cMyBP-C is controlled by positive and negative regulatory coupling between those sites. PKA phosphorylation of cMyBP-C’s N terminus on 3 conserved serine residues is hierarchical and antagonizes phosphorylation by PKCε, and vice versa. In contrast, RSK2 phosphorylation of cMyBP-C accelerates PKA phosphorylation. We used cMyBP-C’s regulatory N-terminal domains in defined phosphorylation states for protein–protein interaction studies with isolated cardiac native thin filaments and the S2 domain of cardiac myosin to show that site-specific phosphorylation of this region of cMyBP-C controls its interaction with both the actin-containing thin and myosin-containing thick filaments. We also used fluorescence probes on the myosin-associated regulatory light chain in the thick filaments and on troponin C in the thin filaments to monitor structural changes in the myofilaments of intact heart muscle cells associated with activation of myocardial contraction by the N-terminal region of cMyBP-C in its different phosphorylation states. Our results suggest that cMyBP-C acts as a sarcomeric integrator of multiple signaling pathways that determines downstream physiological function.

## Significance

Phosphorylation of cardiac myosin binding protein-C (cMyBP-C) is a key regulator of myocardial contractility, and dephosphorylation of cMyBP-C is associated with heart failure. However, the molecular mechanisms underlying contractile regulation by cMyBP-C phosphorylation are poorly understood. We describe the kinase specificity of the multiple phosphorylation sites on cMyBP-C and show that they are interdependent and have distinct effects on the structure of the thin and thick filaments. The results lead to a model of regulation by cMyBP-C phosphorylation through altered affinity of cMyBP-C’s N terminus for thin and thick filaments, as well as their structures and associated regulatory states. Impairment of these mechanisms is likely to underlie the functional effects of mutations in filament proteins associated with cardiomyopathy.

Contraction of cardiac muscle is initiated by activation of the actin-containing thin filaments, but is modulated by structural changes in the myosin-containing thick filaments. Calcium binding to troponin induces an azimuthal movement of tropomyosin on the surface of the thin filaments which allows myosin head domains from the neighboring thick filaments to strongly attach to actin (1). Subsequently, small conformational changes in the actin-attached myosin catalytic domain are amplified by the essential and regulatory light chain-containing myosin light chain domain or “lever arm” associated with the release of Adenosine 5′-triphosphate hydrolysis products (2, 3). This “working stroke” produces piconewton-scale force and nanometer-scale displacement of the thin filaments toward the center of the sarcomere.

Heart muscle contractility is also regulated by posttranslational modifications of sarcomeric proteins, including phosphorylation of the regulatory components of the thick filaments (4). Phosphorylation of these components has been widely implicated in the regulation of cardiac output, and altered phosphorylation levels have been frequently associated with heart failure (5), further underlining their functional significance. In the current study, we focused on phosphorylation of cardiac myosin binding protein-C (cMyBP-C), a thick filament-associated protein with important regulatory functions in both healthy and diseased states of the heart. The functional significance of cMyBP-C phosphorylation is highlighted by the fact that ablation of either cMyBP-C or its phosphorylation leads to pathological hypertrophy in animal models, suggesting that cMyBP-C phosphorylation is essential for normal heart function (6, 7).

**Fig. 1.**
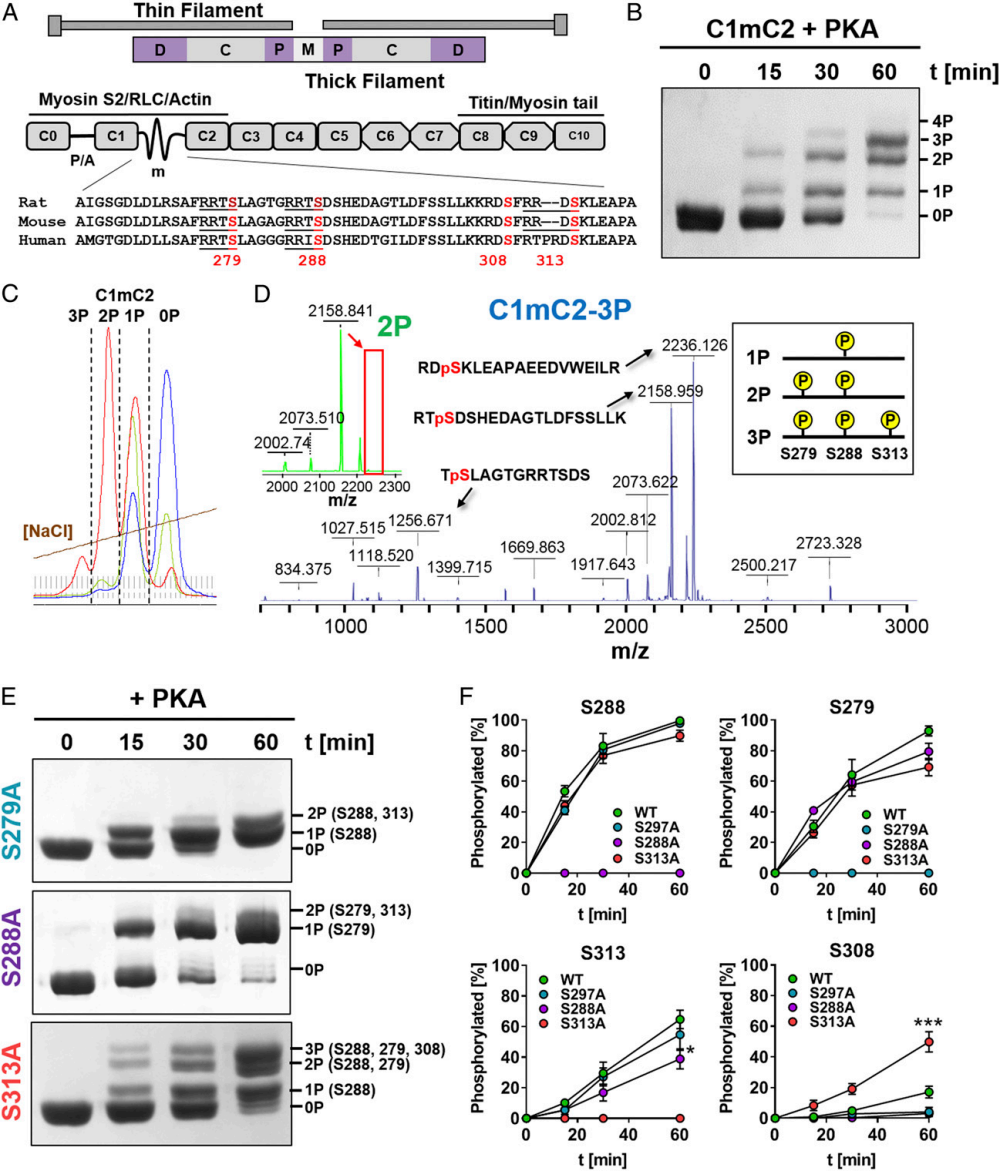
Hierarchical phosphorylation of C1mC2 by PKA. (*A*, *Top*) Diagram of the sarcomere. The thick filament D-, C-, and P-zones and M-line are labeled accordingly. (*A*, *Bottom*) Domain organization and known protein interactions of cMyBP-C. The proline/alanine-rich linker (P/A) between domains C0 and C1 and the m-motif (m) between domains C1 and C2 are labeled accordingly. Sequence alignment of the m-motif residues encompassing the conserved phosphorylation sites (red) is shown below, with canonical PKA consensus sequences (RRXS) underlined. Serines are numbered according to the rat cMyBP-C sequence. (*B*) Time (t)-dependent in vitro phosphorylation of C1mC2 by PKA analyzed by Phos-tag–SDS/PAGE. (*C*) Chromatogram demonstrating the separation of phosphorylated C1mC2 constructs (0P, 1P, 2P, and 3P) by IEC on the Resource S column. Blue, green, and red traces correspond to phosphorylation reactions stopped at different time points. (*D*) MS analysis of phosphorylated residues in IEC fractions shown in *C*. Example MALDI-MS/MS spectra for tris-phosphorylated C1mC2 (C1mC2-3P, blue) and bis-phosphorylated C1mC2 (C1mC2-2P, green) are shown with peaks labeled with their corresponding phosphorylated peptide sequences. Note that the peak at the 2,236.126 mass-to-charge ratio (*m*/*z*) corresponding to S313-phosphorylated peptide found in C1mC2-3P is missing in the C1mC2-2P spectrum (indicated by a red box). The peak at 1,256.671 *m*/*z* is not shown in the C1mC2-2P spectrum for clarity. Interpretation of the MS data is summarized in the box on the right. Details are provided in text. In vitro kinase assays with S-A–substituted C1mC2 analyzed by Phos-tag–SDS/PAGE (*E*) and deconvolution into phosphorylation of individual serine residues (*F*) are shown. Mean ± SEM (*n* = 6). Statistical significance of differences between values was assessed with a one-way ANOVA followed by Tukey’s post hoc test: *P < 0.05, ***P < 0.001.

cMyBP-C is localized to 9 transverse stripes in the central region of each half-thick filament, called the C-zone, via interactions of its C-terminal anchoring region with the myosin tails and titin (Fig. 1*A*), closely matching the ∼43-nm periodicity of the myosin head domains. In contrast, interactions of its regulatory N-terminal domains are less well defined, and binding sites for both myosin and actin have been identified in vitro (8). Myosin interactions of cMyBP-C’s N terminus are generally associated with an inhibitory effect on contractility, and both structural and functional studies suggest that cMyBP-C stabilizes the thick filament OFF state by tethering myosin head domains to the surface of the thick filament backbone (9, 10). In contrast, cMyBP-C’s N-terminal domains have also been shown to bind actin and activate the thin filament presumably by moving tropomyosin away from its blocked position, which increases the calcium sensitivity of its regulatory units (11, 12).

Both the inhibitory and activating interactions of cMyBP-C are believed to be controlled by its phosphorylation state. The cardiac specific “m-motif” between domains C1 and C2 contains a series of conserved serine residues that are phosphorylated by several protein kinases in vivo and in vitro (13) (Fig. 1*A*). Protein kinase A (PKA) has been identified as the primary kinase acting upon cMyBP-C (14), mediating some of the effects of β-adrenergic stimulation on myocardial function, such as increased cross-bridge kinetics, decreased calcium sensitivity, and accelerated relaxation (15, 16). More recently, other protein kinases have been shown to phosphorylate cMyBP-C with distinct specificities for m-motif serines, suggesting that each phosphorylation site might have distinct regulatory effects (13).

However, the detailed function and mechanism underlying cMyBP-C phosphorylation at individual sites remain poorly understood, likely due to the complexity of its interaction with other sarcomeric components, which is further compounded by the multiplicity of signaling pathways acting upon cMyBP-C and its associated phosphorylation states (17). Current mechanistic hypotheses of the effects of cMyBP-C phosphorylation are largely based on in vivo experiments in animal models (6, 18) or tissues derived from those animals (19) in which phosphorylation levels and associated regulatory mechanisms cannot be controlled at the molecular level. Moreover, serine-to-aspartate substitutions have been frequently used as a convenient model for phosphorylation, although recent studies showed that these substitutions only partially recapitulate the effects of phosphorylation (20). Conversely, in vitro experiments with fully phosphorylated proteins or proteins containing serine-to-aspartate substitutions do not recapitulate the in vivo complexity of cMyBP-C’s interactions and its dynamic phosphorylation (21–23).

The aim of the present work was to understand the molecular mechanism of cMyBP-C phosphorylation and its structural and functional effects on both thin and thick filament-based regulation in heart muscle cells. We investigated the relationship of individual cMyBP-C phosphorylation sites and potential cross-talk using several protein kinases known to phosphorylate cMyBP-C. We utilized cMyBP-C fragments with well-characterized phosphorylation states to show that site-specific phosphorylation has distinct effects on its interaction with and regulation of the thin and thick filaments, combining in vitro biochemical binding assays with in situ structural measurements in intact heart muscle cells. The results show that cMyBP-C acts as a sarcomeric integrator of different signaling pathways to determine downstream physiological effects, and that the functional effects of cMyBP-C phosphorylation can only be understood by combining thin and thick filament-based mechanisms into an integrated model of contractile regulation.

## Results

### Hierarchical Phosphorylation of cMyBP-C by PKA.

To elucidate the regulatory function of cMyBP-C phosphorylation, we phosphorylated a recombinant fragment of rat cMyBP-C containing domains C1, the phosphorylatable m-motif, and C2 (C1mC2; Fig. 1*A*) with the catalytic subunit of PKA in vitro (Fig. 1*B*). The C1mC2 fragment is a convenient model for studying the function of full-length cMyBP-C, particularly with respect to its effects on both thin and thick filament structure (11, 12, 20) and its regulation by phosphorylation (20, 24, 25). Although rodent and human C1mC2 have a high sequence identity (>92%), suggesting a conserved molecular function, species-specific effects cannot be excluded (26).

Incubation of C1mC2 for 30 to 60 min with PKA resulted in a mixture of unphosphorylated, monophosphorylated, bis-phosphorylated, and tris-phosphorylated protein, which could be clearly separated by both Phos-tag and sodium dodecyl sulfate polyacrylamide gel electrophoresis (SDS/PAGE) (Fig. 1*B*). Individual C1mC2 phospho-species were isolated by stopping the kinase reaction at different time points and separating the phosphorylated proteins by ion-exchange chromatography (IEC) (Fig. 1*C*). The phosphorylation level and homogeneity (>95%) of each IEC fraction (e.g., 1P, 2P, 3P) were confirmed by electrospray ionization (ESI) mass spectrometry (MS) (*SI Appendix*, Table S1). Tetrakis-phosphorylated C1mC2 was only observed after prolonged incubation with PKA at 30 °C, suggesting that the fourth site is a poor substrate for PKA.

Phosphorylated amino acid residues in each IEC fraction were identified by proteolytic digestion followed by phospho-peptide enrichment and matrix-assisted laser desorption ionization (MALDI) MS and ESI-MS (*SI Appendix*, Fig. S1) (details are provided in *SI Appendix*, *Supplementary Information Methods*). Analysis of the IEC fraction corresponding to monophosphorylated C1mC2 (C1mC2-1P) revealed specific phosphorylation on a single serine residue, S288, in agreement with previous studies suggesting that S288 in the cardiac-specific insertion is the initial PKA phosphorylation site (14). Bis-phosphorylated C1mC2 (C1mC2-2P) contained phosphorylated serine residues only in positions 288 and 279, and tris-phosphorylated C1mC2 (C1mC2-3P) contained phosphorylated serines only in positions 288, 279, and 313. As an example, the MALDI-MS spectrum of C1mC2-3P is shown in Fig. 1*D*, with peaks corresponding to identified phospho-peptides labeled accordingly. The peak at a 2,236.126 mass-to-charge ratio corresponding to the S313-phosphorylated peptide is missing in the MALDI-MS spectrum of the bis-phosphorylated C1mC2 (C1mC2-2P) (Fig. 1 *D*, *Inset*, *Top Left*, red box), suggesting that S313 is phosphorylated after S288 and S279. In contrast, phosphorylation of S308 by PKA was only observed after prolonged incubation, suggesting that this serine is a poor substrate for PKA in vitro. Thus, our results suggest that PKA phosphorylation of the cardiac-specific m-motif follows a concerted hierarchical mechanism in the sequence of S288, followed by S279, followed by S313 (Fig. 1 *D*, *Inset*, *Top Right*).

To further test this conclusion, we prepared serine-to-alanine (S-A) substitutions of each individual PKA site in C1mC2, and analyzed their phosphorylation profiles by Phos-tag–SDS/PAGE (Fig. 1*E*). Total phosphate incorporation after 60 min of incubation was strongly reduced in S288A-substituted C1mC2 (1.25 ± 0.08 mol of inorganic phosphate [P_i_]/mol [mean ± SEM]; *n* = 6; *SI Appendix*, Fig. S2) compared with wild type (2.94 ± 0.18 mol of P_i_/mol [mean ± SEM]; *n* = 6). S279A- and S313A-substituted C1mC2 showed intermediate levels of phosphorylation, although S279A had a stronger inhibitory effect than S313A (1.6 ± 0.11 and 2.17 ± 0.12 mol of P_i_/mol, respectively), in agreement with the hierarchical phosphorylation sequence proposed above.

Next, we analyzed the effects of S-A substitutions on phosphate incorporation by PKA at each individual phosphorylation site (Fig. 1*F*). Consistent with the hierarchical model, phosphorylation of S288 was not significantly affected by substitution of either S279 or S313 by alanine. Similarly, S279 phosphorylation was not inhibited by substitution of either S288 or S313 by alanine, although in the native fragment, this residue is phosphorylated after S288. This suggests that unphosphorylated S288 may be involved in intramolecular interactions that prevent access to S279, and that substitution of S288 by alanine abolishes this interaction. If so, substitution of S288 by alanine might therefore partially mimic phosphorylation of this site. In contrast, S313 phosphorylation was inhibited by substitution of either S288 or S279 to alanine, further supporting the proposal that this residue is phosphorylated downstream of S288 and S279. Contrary to the effects of S-A substitutions described above, PKA phosphorylation of S308 was greatly increased in C1mC2-S313A (∼50%) compared with wild-type control (∼10%), suggesting that phosphorylation of S313 per se has an inhibitory effect on phosphorylation of S308.

### cMyBP-C Phosphorylation by Non-PKA Kinases Reveals Regulatory Coupling of Phosphorylation Sites.

Although PKA is considered to be the primary kinase acting upon cMyBP-C in vivo, several other kinases have been shown to be able to phosphorylate specific residues within the cardiac-specific m-motif (13). Moreover, these kinases have been frequently shown to be activated during heart disease, underlining their functional significance.

**Fig. 2.**
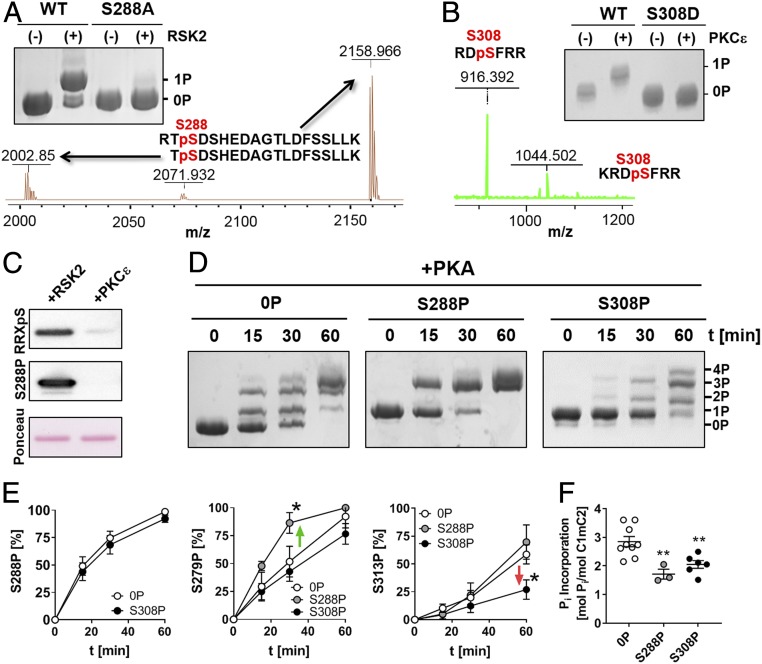
C1mC2 phosphorylation by RSK2 and PKCε. (*A*) RSK2-mediated phosphorylation of S288 in rat C1mC2 was confirmed by in vitro kinase (IVK) assays using wild-type and S288A- substituted C1mC2 as well as MALDI-MS phosphorylation site profiling. *m*/*z*, mass-to-charge ratio. (*B*) PKCε phosphorylation of S308 in rat C1mC2 was confirmed by both IVK assays using phosphoablated C1mC2-S308D and ESI-MS. (*C*) Western blot analysis of RSK2- and PKCε-phosphorylated C1mC2 using PKA site (RRXpS)-specific and S288 (pS288)-specific antibodies. Note that PKCε-phosphorylated C1mC2 is not recognized by either of the antibodies, confirming S308 as the main phosphorylation site. (*D*) PKA IVK assays with unphosphorylated, S288-phosphorylated, or S308-phosphorylated C1mC2 analyzed by Phos-tag–SDS/PAGE. t, time. Analysis of individual site phosphorylation (*E*) and total level of phosphate incorporation by PKA (*F*) are shown. Mean ± SEM (*n* = 3 to 9). Statistical significance of differences between values was assessed with a one-way ANOVA followed by Tukey’s post hoc test: *P < 0.05, **P < 0.01.

Ribosomal S6 kinase II (RSK2) has been suggested to specifically phosphorylate serine residues in the cardiac-specific m-motif insertion in response to activation of the mitogen-activated protein kinase/extracellular signal-regulated kinase pathway (27), and we used site-specific S-A substitution and MS to confirm these results (Fig. 2*A* and *SI Appendix*, Fig. S3). RSK2 primarily phosphorylated rat C1mC2 at S288 in vitro, although we observed phosphorylation of other residues at higher enzyme-to-substrate ratios or after prolonged incubation. In contrast, α-adrenergic stimulation leads to an increase in cMyBP-C phosphorylation primarily via stimulation of protein kinase Cε (PKCε) (28, 29) [and protein kinase D (PKD) (30)] activity. We used site-directed mutagenesis, MS, and Western blot analysis to confirm that PKCε specifically phosphorylates S308 in C1mC2 in vitro (Fig. 2 *B* and *C* and *SI Appendix*, Fig. S3). Phosphorylation of S288 was not a prerequisite for phosphorylation of S308 by PKCε as shown previously (31).

Next, we investigated potential cross-talk between cMyBP-C–mediated phosphorylation by RSK2, PKCε, and PKA using purified C1mC2 fragments site-specifically phosphorylated by RSK2 and PKCε as substrates in PKA kinase assays. As expected from the sequential phosphorylation of C1mC2 described above, S288 phosphorylation by RSK2 significantly accelerated phosphorylation of S279 by PKA, but had no effect on phosphorylation of residues downstream of S279 (i.e., S313) (Fig. 2 *D* and *E*). In contrast, phosphorylation of S308 by PKCε decreased the rate of PKA phosphorylation of S313, suggesting that S308P is a negative regulator of cMyBP-C PKA phosphorylation of S313, consistent with previous results in isolated cardiomyocytes (32). Moreover, both S288 by RSK2 and S308 by PKCε phosphorylation reduced overall phosphate incorporation into C1mC2 by PKA by ∼1 mol of P_i_/mol of C1mC2 (Fig. 2*F*). Although this is expected for phosphorylation of S288 as a main PKA site, S308 is primarily phosphorylated by PKCε and PKD, further confirming the antagonistic effects of PKA- and PKCε/PKD-mediated phosphorylation of cMyBP-C.

In summary, the multiple cMyBP-C phosphorylation sites are not independent, and exhibit positive and negative regulatory coupling with partially overlapping specificity for multiple kinases.

### Site-Specific Phosphorylation Controls Thick and Thin Filament Binding of C1mC2.

To characterize the effects of site-specific cMyBP-C phosphorylation on its proposed interactions with both the myosin-containing thick and actin-containing thin filaments (12, 20), we determined the binding affinities of C1mC2 in its different phosphorylation states for isolated myosin S2 and native thin filaments (NTFs) in vitro. The affinity of C1mC2 for its binding site on myosin, the first 126 amino acids of myosin S2 (S2Δ), was characterized by microscale thermophoresis (MST). As previously shown, unphosphorylated C1mC2 binds S2Δ in a biphasic manner, corresponding to high- and low-affinity binding sites with dissociation constants (K_d_s) of 20.7 ± 3.5 μmol/L (mean ± SEM; *n* = 6) and >400 μmol/L, respectively (20). Moreover, the K_d_ for myosin S2Δ of λ-protein phosphatase–treated native full-length cMyBP-C isolated from rat ventricular tissue was 24.2 ± 3.9 μmol/L (mean ± SEM; *n* = 4) (*SI Appendix*, Fig. S4), in good agreement with the results obtained for recombinant rat C1mC2.

**Fig. 3.**
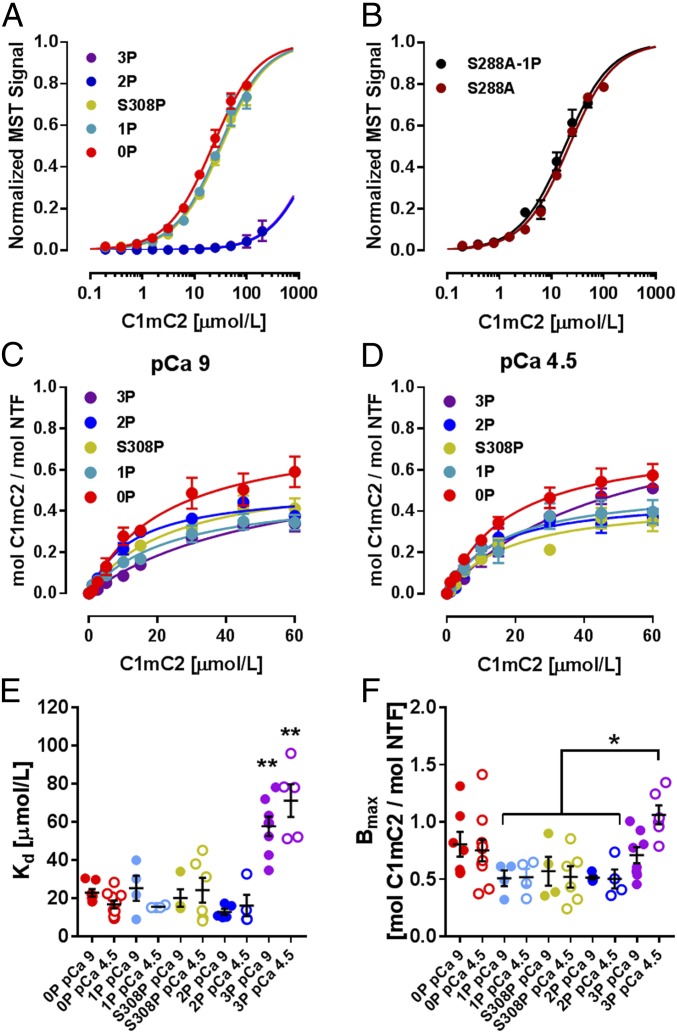
Effects of site-specific phosphorylation on binding of C1mC2 to myosin S2Δ and NTFs. (*A*) Normalized MST curves for C1mC2 and its different phosphorylation states titrated against myosin S2Δ. (*B*) Normalized MST binding curves for C1mC2-S288A (brown) and C1mC2-S288A-1P (black) titrated against myosin S2Δ. NTF cosedimentation data for C1mC2 and its different phosphorylation states in the absence (*C*, pCa 9) and presence (*D*, pCa 4.5) of 32 μmol/L CaCl_2_ are shown. Calculated K_d_ (*E*) and B_max_ (*F*) values for the NTF binding assays in the absence (filled circles) and presence (open circles) of CaCl_2_ are shown. Mean ± SEM (*n* = 4 to 10). Statistical significance of difference was assessed with a one-way ANOVA followed by Tukey’s post hoc test: *P < 0.05, **P < 0.01.

Monophosphorylation of either S308 or S288 slightly reduced C1mC2’s affinity for myosin S2Δ as indicated by an increase in K_d_ to ∼30 μmol/L (Fig. 3*A* and *SI Appendix*, Fig. S5 and Table S2). In contrast, both PKA bis-phosphorylation (S288 and S279) and tris-phosphorylation (S288, S279, and S313) completely abolished S2Δ binding, indicating that either bis-phosphorylation or phosphorylation of S279 per se controls the cMyBP-C–myosin S2 interaction. We addressed this question by phosphorylating C1mC2-S288A with PKA and isolating the monophosphorylated species (C1mC2-S288A-1P) using IEC. Both unphosphorylated and monophosphorylated C1mC2-S288A bind myosin S2Δ with a K_d_ similar to that measured for the wild-type protein (K_d_ of ∼20 μmol/L), suggesting that bis-phosphorylation is necessary and sufficient to abolish cMyBP-C–myosin S2 interaction (Fig. 3*B* and *SI Appendix*, Table S2). To further test this conclusion, we sequentially phosphorylated C1mC2 with RSK2 and PKCε, and measured the affinity of the bis-phosphorylated C1mC2 (S288 and S308) for myosin S2Δ (*SI Appendix*, Fig. S6). The RSK2/PKCε bis-phosphorylated C1mC2 did not bind to myosin S2Δ, further supporting the hypothesis that bis-phosphorylation per se, independent of the phosphorylation site combination, abolishes cMyBP-C–myosin S2 interaction. These results are consistent with the largely ionic nature of cMyBP-C’s main interaction site in the m-motif and myosin S2 (20).

We measured the affinity of C1mC2 in its different phosphorylation states for isolated bovine cardiac NTFs using high-velocity cosedimentation. Unphosphorylated C1mC2 (0P) binds NTFs in the absence of calcium (pCa 9) in a saturable manner with a K_d_ of ∼20 μmol/L and a maximal binding capacity (B_max_) of ∼1, indicating stoichiometric binding of C1mC2 to actin (Fig. 3 *C*–*F* and *SI Appendix*, Fig. S5 and Table S2). Addition of calcium (pCa 4.5) had no effect on C1mC2 binding to NTFs as indicated by identical K_d_ and B_max_ values (Fig. 3 *D*–*F* and *SI Appendix*, Fig. S5*A* and Table S2). Strikingly, both PKA monophosphorylation (S288) and bis-phosphorylation (S288 and S279), as well as monophosphorylation of S308 by PKCε, showed no change in the affinity of C1mC2 for NTFs (K_d_ ∼ 20 μmol/L), but decreased B_max_ to ∼0.5, suggesting a lower binding capacity of C1mC2 in the partially phosphorylated states. As before, full calcium activation of NTFs had no additional effect on either K_d_ or B_max_. However, PKA tris-phosphorylation (3P) significantly decreased C1mC2’s affinity for NTF with an estimated K_d_ of ∼60 μmol/L independent of [Ca^2+^]. Addition of calcium increased B_max_ from ∼0.5 to ∼1 for C1mC2-3P, suggesting an interplay between tris-phosphorylation of C1mC2, thin filament binding, and calcium activation (23).

These results suggest that cMyBP-C phosphorylation regulates contractility partly via differential modulation of its affinity for myosin and actin binding sites, so that phosphorylation leads to a redistribution of cMyBP-C’s N-terminal domains from their myosin binding sites in the thick filaments to their actin binding sites in the thin filaments. Monophosphorylation weakens and bis-phosphorylation abolishes thick filament binding, and only tris-phosphorylation affects C1mC2 binding to regulated thin filaments.

### Site-Specific Phosphorylation of C1mC2 Controls Its Effect on Thin and Thick Filament Structure.

**Fig. 4.**
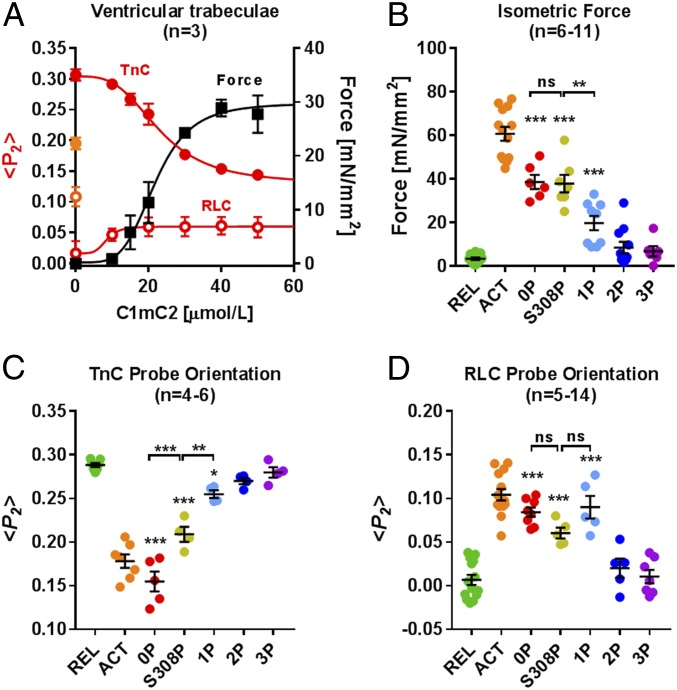
Effects of site-specific phosphorylation of C1mC2 on active force and thin and thick filament structure in ventricular trabeculae. (*A*) Concentration-dependent effect of C1mC2 on force generation (black squares) and thin (red filled circles) and thick (red open circles) filament structure in ventricular trabeculae. TnC and RLC probe orientations are expressed as the order parameter <*P*_*2*_>, which is +1 for a probe dipole orientation parallel to the filament axis and −0.5 for a perpendicular orientation. <*P*_*2*_> for full calcium activation is shown in orange on the left axis. (*B*) Isometric force of ventricular trabeculae in the presence of 40 μmol/L C1mC2 in its different phosphorylation states. ACT, activating solution (pCa 4.5); REL, relaxing solution (pCa 9). TnC (*C*) and RLC (*D*) probe orientations of ventricular trabeculae in the presence of 40 μmol/L C1mC2 in its different phosphorylation states are shown. Mean ± SEM, with the number of trabeculae (n), is indicated in each panel. Statistical significance of differences between groups was assessed with a one-way ANOVA followed by Tukey’s post hoc test: *P < 0.05, **P < 0.01, ***P < 0.001. ns, not significant.

Next, we used a bifunctional rhodamine probe attached to the E-helix of cardiac troponin C (cTnC-E) to monitor structural changes in the thin filaments of demembranated ventricular muscle cells associated with the activating effect of C1mC2 described previously (12). Unphosphorylated C1mC2 activates the force and thin filament structure of ventricular trabeculae in the absence of Ca^2+^ (pCa 9) with a half-maximal effective concentration (EC_50_) of ∼20 μmol/L (Fig. 4*A*, filled red circles). Maximum isometric force at [C1mC2] = 40 μmol/L is only ∼60% of that measured during Ca^2+^ activation in the absence of C1mC2 (Fig. 4*B*), although the level of thin filament activation as reported by the cTnC probe is significantly higher, suggesting that exogenous C1mC2 has both activating and inhibitory effects on contractility.

PKA phosphorylation of S288 reduced the activating effect of 40 μmol/L C1mC2 on active force to ∼30% of that measured during control conditions (Fig. 4*B*, light blue, 1P), to less than 10% after PKA bis-phosphorylation (C1mC2-2P, dark blue, 2P), and active force was completely abolished for PKA tris-phosphorylated C1mC2 (C1mC2-3P) (Fig. 4*B*, purple). In contrast, S308 phosphorylation by PKCε did not inhibit the activating effect of C1mC2 on force generation (Fig. 4*B*, yellow), suggesting different regulatory functions of phosphorylation of S288 and S308.

The effects on isometric force described above are mirrored by those on the thin filament structure as monitored by the cTnC-E probe orientation. C1mC2 “superactivates” the thin filament as indicated by a change in the order parameter <*P*_*2*_>, which is significantly larger than that observed for calcium activation alone (∼120%) (Fig. 4*C*). PKA monophosphorylation reduced the activating effect to ∼30% of that measured during control conditions in the absence of C1mC2, in agreement with the force data. In contrast, S308 monophosphorylated C1mC2 showed an intermediate effect on thin filament activation, corresponding to ∼70% of the control value (Fig. 4*C*, yellow). Incubation of ventricular trabeculae with either 40 μmol/L PKA bis- or tris-phosphorylated C1mC2 had no significant effect on thin filament structure in the absence of Ca^2+^.

These results are in stark contrast to the NTF binding data described above, suggesting that although phosphorylation of cMyBP-C has only minor effects on its binding to the thin filament, it significantly alters thin filament regulation. Recent electron microscopy studies demonstrated that N-terminal domains of cMyBP-C bind polymorphically to isolated actin filaments, and that only a subset of binding modes can directly interfere with tropomyosin’s position and induce the ON state of the thin filament (33, 34). The comparison suggests that cMyBP-C phosphorylation regulates thin filament activation by altering the equilibrium between binding states that affect tropomyosin’s position on actin and those that do not.

Structural changes in the thick filament associated with the activation of ventricular trabeculae by C1mC2 in its different phosphorylation states were monitored using a bifunctional sulforhodamine probe cross-linking helices B and C in the myosin regulatory light chain (BSR-cRLC-BC) (35). BSR-cRLC-BC is localized close to the myosin S1/S2 junction and is mainly sensitive to the regulatory state of the thick filament; the order parameter <*P*_*2*_> from this probe increases upon calcium activation. In contrast to its effect on thin filament structure described above, C1mC2 incubation leads to a partial activation of the thick filament structure corresponding to ∼70% of that measured during control activations (pCa 4.5; Fig. 4*A*, orange open circle). Moreover, C1mC2 activated the thick filament with a significantly lower EC_50_ than that measured for force or thin filament activation (∼10 μmol/L; Fig. 4*A*, open red circles).

Monophosphorylation of either S288 by PKA or S308 by PKCε showed no significant reduction in the activating effect of 40 μmol/L C1mC2 on the thick filament structure as reported by the BC probe orientation (Fig. 4*D*), in contrast to the strong reduction in thin filament activation and isometric force production associated with phosphorylation of S288 (Fig. 4 *B* and *C*; 1P). Thus, although S288 phosphorylation and S308 phosphorylation have similar effects on the regulatory state of the thick filament, they have very different effects on the regulatory state of the thin filament. In agreement with their effects on force and thin filament structure, both C1mC2-2P and C1mC2-3P had no significant effect on thick filament structure as measured by the RLC BC probe orientation, consistent with the abolished binding of C1mC2 to myosin S2Δ after PKA bis-phosphorylation and tris-phosphorylation (Fig. 3*A* and *SI Appendix*, Table S2).

The comparison of the effects of C1mC2 in its different phosphorylation states on the myosin head conformation with the MST binding data described above further suggests that C1mC2 has a direct activating effect on the thick filament and that the activating effect is, in turn, controlled by its phosphorylation-dependent affinity for myosin S2Δ.

### Site-Specific Phosphorylation of C1mC2 Controls Actomyosin ATPase Activity.

**Fig. 5.**
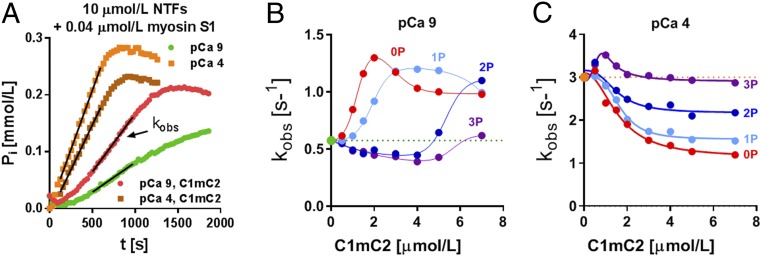
Effect of site-specific phosphorylation of C1mC2 on actomyosin ATPase. (*A*) Effect of C1mC2 on NTF activation in vitro was estimated by measuring the apparent rate constant of steady-state myosin S1-NTF ATPase activity (k_obs_). In the absence of Ca^2+^ (green, pCa 9), the NTF-stimulated myosin S1 ATPase is low (∼0.6 s^−1^), but it significantly increases in the presence of C1mC2 (red). In contrast, at saturating [Ca^2+^] (orange, pCa 4), the NTF-stimulated myosin S1 ATPase activity is high (∼3 s^−1^), but it decreases in the presence of C1mC2 (brown). t, time. The concentration dependence of the effect of C1mC2 in its different phosphorylation states on the myosin S1-NTF ATPase in the absence (*B*) and presence (*C*) of calcium is shown. Data points were fitted to an activation/inhibition model yielding EC_50_ and IC_50_.

We further investigated the functional consequences of site-specific C1mC2 phosphorylation on actomyosin interactions by measuring the NTF-stimulated adenosinetriphosphatase (ATPase) activity of isolated bovine myosin S1 in the presence of C1mC2 in its different phosphorylation states using a colorimetric assay (Fig. 5*A*).

Unphosphorylated C1mC2 activates the NTF-stimulated myosin S1 ATPase in the absence of calcium in a concentration-dependent manner (Fig. 5*B*, red) and data points were fitted to an activation/inhibition model as previously described (36). Low concentrations activate phosphate production with an EC_50_ of ∼1 μmol/L and a Hill coefficient of ∼1.5, suggesting cooperative activation of NTFs by C1mC2. In contrast, [C1mC2] > 2 μmol/L inhibits the ATPase activity with a half-maximal inhibitory concentration (IC_50_) of ∼3 μmol/L. PKA monophosphorylation (Fig. 5*B*, light blue) and bis-phosphorylation (Fig. 5*B*, dark blue) of C1mC2 increased the EC_50_ of the activating effect further to ∼2 and ∼5 μmol/L, respectively, and tris-phosphorylated C1mC2 did not activate actomyosin S1 ATPase within the concentration range tested (Fig. 5*B*, purple). In fact, C1mC2-2P and C1mC2-3P showed an inhibitory effect on the actomyosin S1-ATPase in the concentration range of 1 to 4 μmol/L, presumably by competing with myosin S1 for binding sites on actin. In contrast, during full calcium activation (Fig. 5*C*, pCa 4), C1mC2 largely inhibits myosin S1-NTF ATPase activity with an IC_50_ of ∼2 μmol/L and a maximal inhibition of ∼50% (Fig. 5*C*, red), similar to the values measured in the absence of Ca^2+^ and [C1mC2] > 2 μmol/L. PKA phosphorylation of C1mC2 decreased the amplitude of inhibition without affecting its IC_50_.

The direct comparison with the NTF binding data (Fig. 3 and *SI Appendix*, Table S2) further supports the conclusion that C1mC2 interacts with NTF in different binding modes and that phosphorylation controls the distribution between states with inhibitory and activating effects. Surprisingly, [C1mC2-3P] < 2 μmol/L increased the NTF-stimulated myosin S1 ATPase in the presence of calcium (pCa 4), consistent with its increased binding capacity for NTFs in presence of Ca^2+^ described above and recent evidence for antagonistic effects of phosphorylation and Ca^2+^ on cMyBP-C structure (23).

## Discussion

### cMyBP-C Coordinates Thin and Thick Filament Activation in the Heart.

Multiple conserved phosphorylation sites have been identified in close proximity to each other within cMyBP-C’s regulatory m-motif (13). Moreover, each phosphorylation site is a substrate for a different set of protein kinases, suggesting that cMyBP-C might act as a central signaling hub within the sarcomere, integrating different signaling pathways to control contractile function. Our results strongly support a model in which phosphorylation of cMyBP-C is controlled by regulatory coupling between individual phosphorylation sites, and that different sites and combinations thereof have distinct regulatory functions. According to this model, activation of protein kinases downstream of cellular signaling pathways results in a distinct cMyBP-C phosphorylation pattern that alters heart muscle contractility by modulating both (*i*) the distribution of cMyBP-C’s N-terminal domains (NcMyBP-C) between binding sites in the thin and thick filaments and (*ii*) the structure and associated regulatory state of the filaments.

**Fig. 6.**
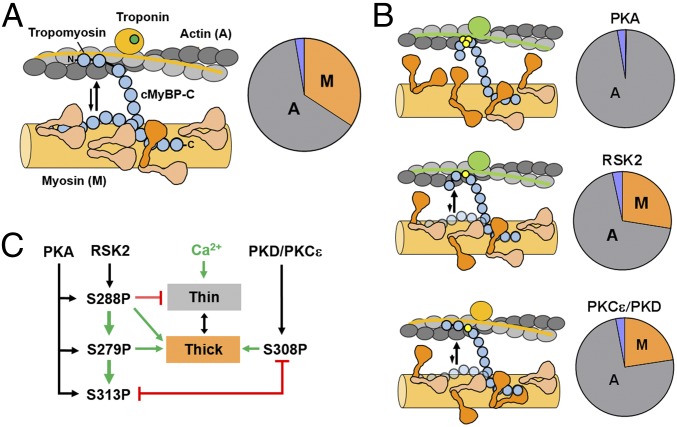
Proposed model for the effects of site-specific cMyBP-C phosphorylation on thin and thick filament-based regulation. (*A*, *Left*) In the dephosphorylated state, some cMyBP-C molecules (blue) are bound to myosin S2 via their N-terminal domains, stabilizing the thick filament OFF state in which the majority of myosin heads (brown) are folded back against the thick filament backbone. The N terminus of other cMyBP-Cs are bound to the thin filaments (gray), which increases the calcium sensitivity of its regulatory units by moving tropomyosin away from its blocked position (troponin/tropomyosin in the ON state are indicated in orange). (*A*, *Right*) Calculated distribution of cMyBP-C’s N-terminal domains bound to either thin (A, gray) or thick (M, orange) filaments. The fraction of unbound cMyBP-C is indicated in purple. (*B*, *Top*) PKA phosphorylation disrupts the cMyBP-C–myosin S2 interaction so that almost all cMyBP-C molecules are bound to the thin filament (gray), which activates the thick filament structure (indicated by brown myosin head domains). PKA phosphorylation also abolishes the sensitizing effect of cMyBP-C on the thin filament by modulating the conformation of cMyBP-C’s N-terminal domains so that its regulatory units are in the OFF state (green ovals depict deactivated troponins). (*B*, *Middle*) RSK2 phosphorylation of S288 partially activates the thick filament, but almost completely inhibits cMyBP-C’s activating effect on the thin filament. (*B*, *Bottom*) PKCε-mediated (or PKD-mediated) phosphorylation of S308 partially activates the thick filament structure by reducing cMyBP-C’s affinity for myosin S2. However, S308 phosphorylation does not inhibit the sensitizing effect of cMyBP-C on the thin filament regulatory units. (*C*) Diagram summarizing the proposed effects of site-specific cMyBP-C phosphorylation on thin (gray) and thick (orange) filament-based regulation, and regulatory coupling between individual phosphorylation sites. The specificity of protein kinases for cMyBP-C phosphorylation sites is indicated by black arrows. Inhibitory and activating effects of individual phosphorylation sites on thin and thick filament regulation are indicated by red and green arrows, respectively. More details are provided in text.

The present results, in agreement with those of a wide range of previous studies, suggest that in the unphosphorylated state, cMyBP-C’s regulatory N-terminal domains interact with both the actin-containing thin and myosin-containing thick filaments with K_d_s in the micromolar range (21, 22, 33). We estimated the effective concentrations of cMyBP-C and of available actin and myosin binding sites in the C-zone using the geometric constraints imposed by the modular architecture of cMyBP-C and the myofilament lattice as about 150, 600, and 300 μmol/L, respectively (*SI Appendix*, *Supplementary Information Text* and Fig. S7). All of the effective concentrations are significantly larger than the measured micromolar K_d_s, suggesting that the fraction of “free” or unbound cMyBP-C in the filament lattice is very low, and that the majority of NcMyBP-Cs are bound to either actin or myosin (Fig. 6 and *SI Appendix*, Fig. S7). Moreover, although C1mC2 binds NTFs and myosin S2Δ with similar affinity, the higher local concentration of available actin binding sites predicts that a larger fraction will be bound to the thin filaments. This conclusion is consistent with electron microscopy reconstructions of resting skeletal muscle demonstrating links between thick and thin filaments with an axial periodicity of ∼43 nm, as expected for MyBP-C (37).

In the current study, we used soluble fragments of rat cMyBP-C’s N-terminal domains as a model system to investigate the structural and functional effects of its phosphorylation on both thin and thick filament-based regulatory mechanisms. Fragment-based experiments do have significant limitations. Truncations might have led to the loss of protein interactions present in full-length cMyBP-C; for example, domain C0 and the P/A-linker, which are not present in the C1mC2 fragment used in the current work, have been shown to enhance the functional effects of cMyBP-C via interaction with both actin and myosin (34, 38). Moreover, exogenous fragments are not subject to the stoichiometric and spatial limitations of endogenous cMyBP-C, and can potentially occupy all possible binding sites within the sarcomere. In contrast, in the intact heart, the effects of cMyBP-C and its phosphorylation are limited to the ∼400-nm-wide C-zone, although they are likely to be communicated to the adjacent D- and P-zones via cooperative interactions in the thin and thick filament structures (12). The former is associated with the end-to-end interaction of tropomyosin molecules between adjacent thin filament regulatory units, and the latter is associated with interactions of myosin head domains in the OFF state (39, 40). Exogenous cMyBP-C fragments might affect myofilament function by direct activating or inhibitory interactions, or by competing with endogenous cMyBP-C for available binding sites, and the current results help to further clarify this issue. We have previously shown that C1mC2 retains its activating effect on the thin filament in the absence of strong binding cross-bridges (12), and the activation of isolated NTF-stimulated myosin S1 ATPase by C1mC2 shows that this effect is not solely mediated by changes in the thick filament structure. These results therefore strongly suggest that both C1mC2 and, by extension, endogenous cMyBP-C have a direct activating effect on the thin filament. In contrast, C1mC2 activates the thick filament, with a significantly lower EC_50_ than that measured for force or thin filament activation (Fig. 5*A*). Moreover, although C1mC2 retains its activating effect on thick filament structure after phosphorylation at either S288 or S308 (Fig. 4*D*), phosphorylation at both sites reduces thin filament activation, and S288 phosphorylation largely abolishes isometric force production of ventricular trabeculae. Taken together, this makes it highly unlikely that C1mC2’s effect on the thick filament is solely mediated via thin filament activation followed by communication of the activation signal from thin to thick filament or through force generation per se, but is rather based on a direct interaction with myosin. This conclusion is consistent with the finding that serine-to-aspartate substitutions in C1mC2 abolish myosin S2Δ binding and reduce thick filament activation without affecting thin filament activation or force generation (20). It follows that C1mC2 fragments that bind to myosin S2Δ have a direct activating effect on the thick filament, independent of any effect on thin filament activation or force generation. It seems therefore more likely that C1mC2’s activating effect on the thick filament is due to a direct competition with the endogenous cMyBP-C, which stabilizes the thick filament OFF state via interactions with the S2 region of myosin, although an alternative mechanism involving a direct effect of C1mC2 on the myosin head domains cannot be excluded by the present results. Therefore, endogenous cMyBP-C and exogenous C1mC2 have the same effect on the thin filament but opposite effects on the thick filament.

We may therefore consider a dephosphorylated state of cMyBP-C, in which about two-thirds of NcMyBP-Cs are bound to the thin filament, locally increasing the calcium sensitivity of its regulatory units in the C-zone (11, 12). The whole thin filament would, however, remain OFF during diastolic conditions due to the low fraction of actins bound to NcMyBP-C. In contrast, the remaining one-third of NcMyBP-Cs are bound to available myosin binding sites, and the consequence of this mode of action of cMyBP-C is the stabilization of the thick filament OFF state, characterized by myosin head domains arranged in helical tracks on the thick filament surface (9) (Fig. 6*A*). The largely calcium-independent affinity of C1mC2 for NTFs further suggests that NcMyBP-C’s interactions with thin filaments are retained during systolic calcium activation. According to this concept, systolic increase in intracellular calcium concentration ([Ca^2+^]_i_) would first activate thin filament regulatory units opposite the C-zone, allowing individual myosin head domains to strongly attach to actin. However, most myosin heads would remain OFF due to inhibitory interactions of NcMyBP-C with myosin S2. As more and more thin filament regulatory units in the C-zone are switched ON, the fraction of actin-attached cross-bridges increases, and a combination of structural changes in the myosin head domains and cMyBP-C would trigger the activation of additional myosin heads. At higher [Ca^2+^]_i_, the rest of the thin filaments would become activated (12). According to this hypothesis, the C-zone itself acts as a sarcomeric signaling element that coordinates thin and thick filament activation, and therefore myocardial contractility.

### PKA Phosphorylation of cMyBP-C Activates Thick Filaments and Inhibits Thin Filaments.

The heart’s inotropic and lusitropic response to β-adrenergic receptor stimulation is, in part, mediated by PKA phosphorylation of cMyBP-C on key sites within the cardiac-specific m-motif (14). The present results strongly suggest that PKA sequentially phosphorylates these sites within cMyBP-C so that the strength of the β-adrenergic stimulus correlates with its phosphorylation profile. Although the structural basis for this concerted mechanism of phosphorylation is currently unknown, it is likely that phosphorylation of the initial site changes the conformation of subsequent sites so that they are better recognized by the kinase. Consistent with this hypothesis, a structural reorganization of cMyBP-C’s N-terminal domains upon PKA phosphorylation has been observed by both electron microscopy (23) and site-directed spectroscopy (41).

PKA monophosphorylation weakens and bis-phosphorylation abolishes C1mC2–myosin S2Δ interactions, suggesting that S279 is the most important PKA site for regulating cMyBP-C’s interaction with myosin S2 without, however, affecting its affinity for NTFs. Thus, PKA phosphorylation promotes thin filament binding of cMyBP-C by breaking the thick filament interaction of its N-terminal domains with myosin S2, associated with a release of myosin head domains from the filament backbone and activation of the thick filament (9, 15, 42) (Fig. 6 and *SI Appendix*, Fig. S8). The functional consequence is a faster systolic activation of contraction, because myosin heads are no longer constrained by cMyBP-C and can bind to activated thin filament regulatory units. According to our model, even a modest reduction in the affinity of the m-motif for myosin S2 via PKA monophosphorylation is sufficient to cause reorientation of a significant fraction of myosin-bound cMyBP-Cs (∼30%) toward their actin binding sites. Although those represent a small fraction of cMyBP-C molecules, we have previously presented evidence that the regulatory transition in the thick filaments is highly cooperative, so that activation of a small fraction of myosin heads (<20%) can activate the whole thick filament structure (35).

In contrast, the redistribution of cMyBP-C’s N-terminal domains toward the thin filament is believed to locally stabilize its ON state, increase myocardial calcium sensitivity in vitro (11, 12, 36), and prolong the ejection phase and impair relaxation in animal models in vivo (43). In contrast to the phosphorylation-dependent interaction with myosin S2, the largely phosphorylation-insensitive interaction of C1mC2 with NTFs and the high local concentration of available actin binding sites suggests that almost all of the PKA bis-phosphorylated and tris-phosphorylated cMyBP-C will be bound to the thin filaments (Fig. 6*B*). However, PKA phosphorylation inhibits C1mC2’s activating effect on thin filament structure (Fig. 4*C*), implying that increased thin filament binding of cMyBP-C upon phosphorylation is counteracted by a simultaneous weakening of its activating effect, reducing calcium sensitivity (15) and facilitating relaxation (44). In contrast to the intermediate effect on the thick filament described above, monophosphorylation is sufficient to reduce the activating effect of C1mC2 to ∼30% of that measured for unphosphorylated C1mC2 (Fig. 4*C*), suggesting that S288 is the main regulator of cMyBP-C’s effect on thin filament activation. Bis-phosphorylation further reduces (to ∼10%) and tris-phosphorylation completely abolishes C1mC2’s activating effect on thin filament structure as measured by cTnC probe orientation. This nonlinear response to cMyBP-C phosphorylation is consistent with the progressive decrease in C1mC2’s ability to activate NTF-stimulated myosin S1 ATPase after sequential phosphorylation of the 3 PKA sites (Fig. 5*B*).

Taken together with the hierarchical order of phosphorylation sites discussed above, these results suggest that β-adrenergic stimulation mainly reduces thin filament calcium sensitivity via PKA phosphorylation of S288, which subsequently facilitates phosphorylation of S279 and activation of the thick filament (Fig. 6).

Serine 288 is also phosphorylated by RSK2 (Fig. 2*A*), and it was previously shown that RSK2-mediated in situ phosphorylation of cMyBP-C in skinned ventricular trabeculae increases cross-bridge kinetics and decreases calcium sensitivity (27), consistent with the S288-mediated partial activation of the thick filament structure and inhibition of the thin filament structure proposed here (Fig. 6*B*).

In summary, cMyBP-C exists in different regulatory states depending on its phosphorylation profile, suggesting that cardiac myofilament function is fine-tuned by the relative distribution of cMyBP-C between its different phosphorylation states (e.g., 0P, 1P, 2P, 3P). According to this model, even moderate changes in basal levels of cMyBP-C phosphorylation would have significant functional consequences for the myocardium.

### PKCε Phosphorylation of cMyBP-C Increases Thick Filament Activation without Inhibiting the Thin Filament.

In contrast, phosphorylation of S308 does not abolish the activating effect of C1mC2 on thin filament or force development, but similarly reduces its affinity for myosin S2Δ. Thus, phosphorylation of S308 in cMyBP-C is predicted to partially activate the thick filament C-zone without the associated deactivation of the thin filament structure (Fig. 6*B*). The consequence would be significantly faster systolic activation of contraction, since myosin head domains would be readily available for interaction with the calcium-activated thin filament regulatory units inside the C-zone. Both PKCε and PKD have been shown to phosphorylate S308 in vivo and in vitro, suggesting a direct link between α-adrenergic receptor stimulation (45, 46), S308 phosphorylation, and the inotropic response of the heart. Consistent with this idea, PKD phosphorylation of trabeculae from transgenic mouse lines expressing S22A/S23A-substituted cardiac troponin I showed an increase in cross-bridge kinetics, without an associated decrease in calcium sensitivity (30).

CamKII has been shown to be an important regulator of cMyBP-C function, and, recently, CamKII-mediated phosphorylation of S308 has been implicated in the positive force-frequency relation of cardiac muscle (47), suggesting that the molecular pathway described above for S308 phosphorylation by PKCε might also be triggered by CamKII.

### Functional Implications for Pathophysiology of Contractile Regulation in the Heart.

The phosphorylation-dependent interactions of cMyBP-C have important implications for the physiology and pathophysiology of contractile regulation in the heart, and the current results show that cMyBP-C functions as an integrator of multiple signaling elements that mediate context-specific functions of the myocardium in health and disease. Dephosphorylation of cMyBP-C has been frequently observed during heart failure (17), likely associated with myocardial β-adrenergic receptor desensitization, and the present results suggest that the depressed force-generating capacity and impaired relaxation are, in part, mediated by dephosphorylated cMyBP-C stabilizing the thick and thin filament OFF and ON states, respectively.

Of particular interest in the heart failure setting is the RSK2-mediated phosphorylation of S288, which partially mimics the structural and functional effects of β-adrenergic stimulation and primes S279 for phosphorylation by PKA (Fig. 2*D*) (and potentially by other kinases [e.g., CamKII]). Similar to RSK2, α-adrenergic receptor stimulation has been proposed as an alternative pathway to unlock the inotropic reserve of the failing heart (45), and our results suggest that PKD/ PKCε-mediated phosphorylation of cMyBP-C increases contractile force via direct activation of the thick filament. Although either RSK2 or PKD/PKCε phosphorylation only partially mimics the effects of β-adrenergic signaling, phosphorylation by both kinases mimics PKA bis-phosphorylation and abolishes the C1mC2–myosin S2Δ interaction, suggesting that the combination of both pathways might constitute a more effective heart failure treatment.

The concept of modulating the distribution of cMyBP-C’s N-terminal domains between inhibitory binding sites in the thick filaments and activating binding sites in the thin filaments has wider implications for the regulation of cardiac contractility. Other signaling pathways such as RLC phosphorylation or length-dependent activation, the cellular analog of the Frank–Starling law of the heart, might act through a similar mechanism by disrupting cMyBP-C–myosin interactions and favoring binding of the N-terminal domains of cMyBP-C to the thin filament. From the perspective of the well-known mechanisms of cardiac muscle regulation, the current results therefore require a paradigm shift that integrates both thin and thick filament-based mechanisms into a single model of contractile regulation, with a key role for cMyBP-C.

## Methods

Protein production and phosphorylation, preparation of cardiac trabeculae, protein exchange protocols, and fluorescence polarization experiments were performed according to published protocols. Details of materials and methods are provided in *SI Appendix*, *Supplementary Information Methods*.

## Supplementary Material

Supplementary File
