# Supplementary material for: Site-specific phosphorylation of myosin binding protein-C coordinates thin and thick filament activation in cardiac muscle
Source: Proc Natl Acad Sci U S A. 2019 Jul 15;116(31):15485–94. doi: 10.1073/pnas.1903033116 (PMC6681757; doi:10.1073/pnas.1903033116)
Supplement: Supplementary File [file pnas.1903033116.sapp.pdf]

PNAS
www.pnas.org

Supplementary Information for

Site-specific phosphorylation of myosin binding protein-C coordinates thin and thick filament activation in cardiac muscle

Saraswathi Ponnam, Ivanka Sevrieva, Yin-Biao Sun, Malcolm Irving and Thomas Kampourakis

Thomas Kampourakis
Email: thomas.kampourakis@kcl.ac.uk

This PDF file includes:

Supplementary text
Figures S1 to S7
Tables S1 to S2
References for SI reference citations

Supplementary Information Text

Estimating the effective concentration of cMyBP-C, actin and myosin. We estimated the effective concentration of cMyBP-C by assuming that its C-terminal domains are tightly bound to the thick filament backbone in an axial configuration (1, 2) and that the remaining domains can radially extend from the thick filament backbone (Fig. S7). It was previously shown by electron microscopy (EM) that cMyBP-C contains two hinge points separating the molecule into three segments (3). The C-terminal segment (C6C10) with a length of ~20 nm contains the titin and myosin rod binding sites, and is separated from the ~15 nm central segment (mC2C3C4) via a hinge in domain C5. The N-terminal segment with a length of ~8 nm consists of domains C1 and C0, and is separated from the central segment by a hinge in the m-motif linker. Moreover, SAXS studies suggested that the N-terminal domains C0 through C2 adopt an extended conformation in solution with an average length of ~15 nm (4), in good agreement with the estimated length from the EM images (8 nm C0C1 + 5 nm m-motif + 4 nm C2 ≈ 17 nm). From this we can estimate an average distance of ~22 nm between the N-terminal end of domain C1, and the C-terminal end of the hinge in domain C5 between the central and C-terminal segment. Therefore cMyBP-C's N-terminal domain C1 must be within a quarter sphere with a radius of ~22 nm on the thick filament backbone surface, assuming that conformational constraints do not allow the central segment to bend back towards the C-terminal segment through less than 90° (Fig. S7b). This calculation gives a rough estimate of the local cMyBP-C concentration of about ~150 μmol/L (cMyBP-C's N-terminal domains can occupy a volume of $4/12*\pi*(22\ \text{nm})^3 = 11150\ \text{nm}^3$).

Next we placed myosin head pair crowns onto the thick filament surface according to periodicities and axial spacings estimated from electron microscopy reconstructions of isolated human2 and mouse thick filaments (5). Within the volume occupied by a single molecule, cMyBP-C's N-terminus can make contact with up to two myosin head pairs on two preceding myosin head crowns, giving an efficient concentration of myosin S2 of 2*[cMyBP-C] ≈ 300 μmol/L. Interestingly, our model suggests that NcMyBP-C cannot bind to myosin S2 in myosin head crowns within the same axial level as its C-terminal domains, consistent with the higher mobility of one out of three myosin heads crowns within each 43 nm repeat (2, 5).

To estimate the efficient concentration of actin binding sites, we equally distributed actin filaments around the thick filament with a thick-to-thin filament centre-to-centre distance of 27 nm, revealing that a single cMyBP-C can make contact with up to two thin filaments. Moreover, the comparison of the thick-to-thin filament surface distance with the length of the central and N-terminal segment of cMyBP-C suggests that cMyBP-C's N-terminus can only bind to distinct actin monomers with roughly similar axial position as its C-terminal anchoring region. Therefore depending on the azimuthal orientation of the thin filaments with respect to the thick filament surface, cMyBP-C can interact on average with two actin monomers per thin filament. The efficient concentration of actin can therefore be estimated as following: 2*filaments*2actin monomers*[cMyBP-C] ≈ 600 μmol/L.

Supplementary Information Methods

Protein production and phosphorylation. C1mC2 and myosin S2Δ (first 126 amino acids of myosin S2) were cloned, expressed and purified as described previously (6). Serine-to alanine mutations were introduced into C1mC2 by site-directed mutagenesis. C1mC2 (40 μmol/L) was partially phosphorylated with 2000 U/mL PKA in assay buffer (composition in mmol/L: 20 Tris-HCl, 50 NaCl, 2 $MgCl_2$, 0.1 EDTA, 1 DTT) at 30°C. Reactions were either quenched with SDS-PAGE loading buffer and denaturation for 2 min at 100°C for PhostagTM-SDS-PAGE analysis (7), or by addition of 5 mmol/L EDTA for separation of phospho-species by ion-exchange chromatography (IEC) on MonoS columns (GE Healthcare). RSK2 phosphorylation was performed in assay buffer (composition in mmol/L: 50 Tris-HCl, 10 MgCl2, 1 DTT, 0.1% (v/v) Nonidet-40) with an RSK2 concentration of 1.3 U/mL at 30°C for 30-60 min. PKCε

phosphorylation was performed in assay buffer (composition in mmol/L: 20 HEPES/KOH, 2 MgCl2, 1 DTT, 0.03% (v/v) Triton X-100, 10% (v/v) lipid activator (Calbiochem)) with a PKCε concentration of 2 U/mL at 30°C for 60-90 min. Kinases were purchased from Calbiochem and stored according to manufacturer's instructions.

Partially phosphorylated C1mC2 constructs were diluted five-times in 20 mmol/L MOPS pH 6, loaded onto pre-equilibrated MonoS column and proteins eluted with a linear gradient of 0-200 mmol/L NaCl over 30 column volumes. Fractions were analysed by Phostag-SDS-PAGE and electron spray ionization (ESI) mass spectrometry, and fractions showing >95% homogeneity pooled. Pooled protein fractions were concentrated to over 100 μmol/L and stored at -80°C for experiments. Protein and phosphorylation site purity of C1mC2 fragments were estimated to >95% by mass spectrometry and Phostag^{TM}-SDS-PAGE.

Native cMyBP-C was purified from rat cardiac myofibrils by extraction in buffer (composition in mmol/L: 10 EDTA, 124 NaH_2PO4, 31 Na_2HPO4, pH 5.9) followed by protein precipitation in the presence of 55% saturated ammonium sulphate. Precipitated crude cMyBP-C was resolubilized in 20 mmol/L Tris-HCl pH 8.0 containing 0.1 mM EDTA, loaded onto a DEAE-sepharose column and eluted with a linear gradient to 500 mmol/L NaCl. Protein-containing fractions were pooled, concentrated using spin concentrators (AMICON, 30 kDa cutoff) and further purified by gel filtration on a Superose 12 column in PBS containing 10% (v/v) glycerol.

Mass spectrometry analysis. Mass spectrometry analysis was performed by the mass spectrometry service of the University of York (United Kingdom). Purified phosphorylated proteins were run on SDS-PAGE, and gel bands were excised and split in two for parallel in-gel trypsin and Lys-C digestion. Proteolysis was performed after reduction with dithioerythritol and S-carbamidomethylation with iodoacetamide. Gel pieces were washed two times with aqueous 50% (v/v) acetonitrile containing 25 mmol/L ammonium bicarbonate, then once with acetonitrile and dried in a vacuum concentrator for 20 min. Digestion was performed with the addition of 0.2 μg sequencing-grade protease, either modified porcine trypsin (Promega) or Lysobacter enzymogenes endoproteinase Lys-C (Sigma). Proteolysis proceeded overnight at 37°C before acidification with trifluoroacetic acid to stop the reaction. Trypsin and Lys-C digests were then combined before phosphopeptide enrichment using titanium dioxide, as detailed by Larson (8).

A 1 μL aliquot of each enriched peptide mixture was applied directly to a ground steel MALDI target plate, followed immediately by an equal volume of a freshly-prepared 10 mg/mL solution of 2,5-dihydroxybenzoic acid (Sigma) in 50% (v/v) aqueous acetonitrile containing 1% (v/v) phosphoric acid. Positive-ion MALDI mass spectra were obtained using a Bruker ultraflex III in reflectron mode, equipped with a Nd:YAG smart beam laser. MS spectra were acquired over a mass range of m/z 800-4000. Final mass spectra were externally calibrated against an adjacent spot containing 6 peptides (des-Arg1-Bradykinin, 904.681; Angiotensin I, 1296.685; Glu1-Fibrinopeptide B, 1570.677; ACTH (1-17 clip), 2093.086; ACTH (18-39 clip), 2465.198; ACTH (7-38 clip), 3657.929.). Peptide precursors were manually selected for MS/MS fragmentation performed in LIFT mode without the introduction of a collision gas. The default calibration was used for MS/MS spectra, which were baseline-subtracted and smoothed (Savitsky-Golay, width 0.15 m/z, cycles 4); monoisotopic peak detection used a SNAP averaging algorithm (C 4.9384, N 1.3577, O 1.4773, S 0.0417, H 7.7583) with a minimum S/N of 6. Laser power and the number of summed spectra were manually adjusted for each precursor to optimise spectral quality. Bruker flex Analysis software (version 3.3) was used for spectral processing and peak list generation.

Enriched peptide mixtures were loaded onto a nanoAcquity UPLC system equipped with a PepMap 100 Å C18, 5 μm trap column (300 μm x 5 mm Thermo) and an Acclaim PepMap RSLC, 2 μm, 100 Å, C18 RSLC nanocapillary column (75 μm x 150 mm, Thermo). Separation used a gradient elution of two solvents (solvent A: aqueous 1% (v/v) formic acid; solvent B: aqueous 80% (v/v) acetonitrile containing 1% (v/v) formic acid). The flow rate for the capillary column was 300

nL/min. Column temperature was 65°C and the gradient profile was: linear 3-10% B over 7 mins, linear 10-35% B over 30 mins, linear 35-99% B over 5 mins then proceeded to wash with 99% solvent B for 4 min. The column was returned to initial conditions and re-equilibrated for 15 min before subsequent injections. The nanoLC system was interfaced with a maXis HD LC-MS/MS system (Bruker Daltonics) with CaptiveSpray ionisation source (Bruker Daltonics). Positive ESI-MS and MS/MS spectra were acquired using AutoMSMS mode. Instrument control, data acquisition and processing were performed using Compass 1.7 software (microTOF control, Hystar and DataAnalysis, Bruker Daltonics). Instrument settings were: ion spray voltage: 1,450 V, dry gas: 3 L/min, dry gas temperature 150°C, ion acquisition range: m/z 150-2,000, MS spectra rate: 5 Hz, MS/MS spectra rate: 7 Hz at 2,500 cts to 27 Hz at 250,000 cts, cycle time: 1 s, quadrupole low mass: 300 m/z, collision RF: 1,400 Vpp, transfer time 120 ms. The collision energy and isolation width settings were automatically calculated using the AutoMSMS fragmentation table, absolute threshold 200 counts, preferred charge states: 2 – 4, singly charged ions excluded. A single MS/MS spectrum was acquired for each precursor and former target ions were excluded for 0.8 min unless the precursor intensity increased fourfold. Bruker DataAnalysis software (version 4.4) was used for spectral processing and peak list generation.
Tandem mass spectra were searched against an in-house database containing the expected protein sequence using a locally-running copy of the Mascot program (Matrix Science Ltd., version 2.5.1), through the Bruker ProteinScape interface (version 2.1). Search criteria specified: Enzyme, trypsin; Max missed cleavages, 3; Fixed modifications, Carbamidomethyl (C); Variable modifications, Oxidation (M), Phospho (S,T,Y); Peptide tolerance, 10 ppm for LC-MS and 100 ppm for MALDI-MS; MS/MS tolerance, 0.1 Da for LC-MS/MS and 0.5 Da for MALDI-MS/MS; Instrument, ESI-QUAD-TOF or MALD-TOF/TOF. Peptide expect scores and phosphorylation site localisation probabilities were taken from the Mascot search result. Mascot's site localisation probabilities are derived from delta scores as detailed by Savitski (9).

Protein labelling. Myosin S2Δ was labelled with Alexa647-NHS (ThermoScientific) according to manufacturer's instructions. Dye incorporation (>80% efficiency) was confirmed by ESI mass spectrometry. Rat native cMyBP-C was labelled with ATTO647N-maleimide (Molecular Probes) according to manufacturer's instructions. Labelling efficiency after purification was estimated by UV/VIS absorbance spectroscopy to ~1.9 mol ATTO647N/mol cMyBP-C.

Microscale thermophoresis and native thin filament co-sedimentation. Preparation of bovine native thin filaments (NTF), and Microscale Thermophoresis and co-sedimentation experiments were performed as described previously (6).

Native thin filament-myosin S1 ATPase assay. Bovine cardiac myosin S1 (Cytoskeleton Inc.), NTFs and C1mC2 were dialysed and/or gel-filtered into assay buffer (composition in mmol/L: 15 PIPES, 5 MgCl2, and 1 DTT) containing either 1 mmol/L EGTA (pCa 9) or 100 μmol/L $CaCl_2$ (pCa 4). Final concentrations of NTFs and myosin S1 for ATPase assays were fixed at 10 μmol/L and 0.036 μmol/L, respectively. C1mC2 was added in the concentration range between 0-8 μmol/L and the mixture equilibrated at 25°C for 5 min. The reaction was started by addition of ATP to a final concentration of 1 mmol/L in 100 μL final assay volume. ATPase activity was monitored on a real time basis using ATPase ELIPA Biochem Kit (Cytoskeleton Inc.) according to manufacturer's instructions. Assays were conducted in a 96-well format by reading the absorbance at 360 nm every 30 s for a duration of 30 min using a ClarioStar Plate reader system (BMG Labtech). Absorbance values were transformed into phosphate concentrations using phosphate standards and the rate constant of ATP hydrolysis (k_{obs}) determined by linear regression.

Preparation of ventricular trabeculae and fluorescence polarization experiments. Bifunctional rhodamine labelled cTnC (BR-cTnC-E) and bifunctional sulforhodamine labelled cRLC (BSR-cRLC-BC) were prepared as previously described (10, 11).
All animals were treated in accordance with the guidelines approved by the UK Animal Scientific procedures Act (1986) and European Union Directive 2010/63/EU. Wistar rats (male, 200-250 g) were sacrificed by cervical dislocation without the use of anesthetics (Schedule 1 procedure in accordance with UK Animal Scientific Procedure Act, 1986) and demembranated right ventricular trabeculae were prepared as described previously10. BR-cTnC-E was reconstituted into demembranated trabeculae by overnight soak in relaxing buffer (composition in mmol/L: 25 Imidazole, 15 Na2Creatine phosphate (Na_2CrP), 78.4 KPropionate (KPr), 5.65 Na2ATP, 6.8 MgCl2, 10 K_2EGTA, 1 DTT, pH 7.1) containing 0.5 mg/ml of BR-cTnC-E at 4°C, replacing about 80% of the endogenous cTnC (12). BSR-cRLC-BC was exchanged into demembranated trabeculae by extraction in CDTA-rigor solution (composition in mmol/L: 5 CDTA, 50 KCl, 40 Tris-HCl pH 8.4, 0.1% (v/v) Triton X-100) for 30 min followed by reconstitution with 40 µmol/L BSR-cRLC-E in relaxing solution for 1h, replacing ~50% of the endogenous cRLC (13).
Composition of experimental solutions and activation protocols were identical to those described previously for fluorescence polarization experiments (10, 13, 14). Polarized fluorescence intensities were measured as described previously for skeletal and cardiac muscle fibres (10, 13, 15, 16). Fluorescence emission from BSR-cRLC-BC and BR-cTnC-E in trabeculae was collected by a 0.25 N.A. objective using an excitation light beam in line with the emission path. The polarization of the excitation and emitted beams was set either parallel or perpendicular to the trabecular axis, allowing determination of the order parameter $\langle P_2 \rangle$ that describes the dipole orientations in the trabeculae (17).
The sarcomere length of trabeculae was measured by laser diffraction in relaxing solution prior to each activation. Activating solution contained (in mmol/L): 25 Imidazole, 15 Na2CrP, 58.7 KPr, 5.65 Na_2ATP, 6.3 $MgCl_2$, 10 $CaCl_2$, 10 K_2EGTA, 1 DTT, pH 7.1. Each activation was preceded by a 2-min incubation in pre-activating solution (composition in mmol/L: 25 Imidazole, 15 Na_2CrP, 108.2 KPr, 5.65 Na_2ATP, 6.3 $MgCl_2$, 0.2 K_2EGTA, 1 DTT, pH 7.1).
For experiments, C1mC2 constructs were gel-filtered into relaxing solution, concentrated using spin concentrators (AMICON, 10 kDa cutoff) and protein concentrations determined by Bradford assay using a BSA standard.

Statistical analysis. All data sets were normally distributed as assessed by Shapiro-Wilk-test ($P>0.05$). Statistical significance of difference between groups was assessed with a one-way ANOVA followed by Tukey's post-hoc test. Details of significant values are shown in the corresponding figure captions.

a

C1mC2

```
         10         20         30         40         50         60         70         80
GSSMDDPIGL FLMRPQDGEV TVGGSIVFSA RVAGASLLKP PVVKWFKGKW VDLSSKVGQH LQLHDSYDRA SKVYLFELHI
         90        100        110        120        130        140        150        160
TDAQATSAGG YRCEVSTKDK FDSCNFNLTV HEAIGSGDLD LRSAFRRTSL AGTGRRTSDS HEDAGTLDFS SLLKKRDSFR
        170        180        190        200        210        220        230        240
RDSKLEAPAE EDVWEILRQA PPSEYERIAF QHGVTDLRGM LKRLKGMKHD EKKSTAFQKK LEPAYQVNKG HKIRLTVELA
        250        260        270        280        290        300
DPDAEVKWLK NGQEIQMSGS KYIFESIGAK RTLTISQCSL ADDAAYQCVV GGEKCSTELF VKE
```

b

C1mC2-1P

Mass (Da)	Peptide	Position	Best Expect Score	Phosphorylation Position	Localisation Probility (%)	Technique
2001.86	TSDSHEDAGTLDFSSLLK or TSDSHEDAGTLDFSSLLK	137-154	2.00E-07	T137 or S138	48.5 (equal for T137 or S138)	MALDI-MS/MS
2157.96	RTSDSHEDAGTLDFSSLLK or RTSDSHEDAGTLDFSSLLK	136-154	7.40E-07	T137 or S138	51.9 (S138) or 40.4 (T137)	MALDI-MS/MS
2235.04	RDSKLEAPAEEDVWEILR	161-178	1.30E-01	S163	100 - Only one S, T or Y	MALDI-MS/MS

C1mC2-2P

Mass (Da)	Peptide	Position	Best Expect Score	Phosphorylation Position	Localisation Probility (%)	Technique
997.47	RTSLAGTGR	127-135	1.10E-03	S129	50	LC-MS/MS
841.37	TSLAGTGR	128-135	3.80E-02	S129	57	LC-MS/MS
2001.86	TSDSHEDAGTLDFSSLLK	137-154	2.50E-09	S138	61	MALDI-MS/MS
2157.96	RTSDSHEDAGTLDFSSLLK	136-154	3.20E-05	S138	79	MALDI-MS/MS
2157.96	RTSDSHEDAGTLDFSSLLK or RTSDSHEDAGTLDFSSLLK	136-154	1.30E-06	T137 or S138	19 (equal for T137 or S138)	LC-MS/MS
2001.86	TSDSHEDAGTLDFSSLLK or TSDSHEDAGTLDFSSLLK	137-154	3.50E-08	T137 or S138	33.5 (equal for T137 and S138)	LC-MS/MS
2235.06	RDSKLEAPAEEDVWEILR	161-178	2.50E-01	S163	100 - Only one S, T or Y	MALDI-MS/MS

C1mC2-3P

Mass (Da)	Peptide	Position	Best Expect Score	Phosphorylation Position	Localisation Probility (%)	Technique
997.47	RTSLAGTGR	127-135	2.40E-04	S129	73	LC-MS/MS
841.37	TSLAGTGR	128-135	3.80E-03	S129	88	LC-MS/MS
2001.86	TSDSHEDAGTLDFSSLLK	137-154	5.40E-10	S138	62	MALDI-MS/MS
2157.96	RTSDSHEDAGTLDFSSLLK	136-154	5.70E-05	S138	70	MALDI-MS/MS
2157.96	RTSDSHEDAGTLDFSSLLK	136-154	1.60E-07	S138	42	LC-MS/MS
2001.86	TSDSHEDAGTLDFSSLLK or TSDSHEDAGTLDFSSLLK	137-154	9.10E-06	T137 or S138	37 (equal for T137 or S138)	LC-MS/MS
2235.06	RDSKLEAPAEEDVWEILR	161-178	7.80E-09	S163	100 - Only one S, T or Y	MALDI-MS/MS
2235.06	RDSKLEAPAEEDVWEILR	161-178	2.60E-01	S163	100 - Only one S, T or Y	LC-MS/MS

Fig. S1. Mass spectrometry analysis of PKA phosphorylated C1mC2. (a) Rat C1mC2 primary sequence with proposed PKA phosphorylation sites highlighted in red. (b) Phosphorylation site identification of purified C1mC2 phospho-species (1P, 2P and 3P) by LC-MS/MS and MALDI-MS/MS. The numbering of phosphorylated positions corresponds to the amino acid position in C1mC2 shown in (a).

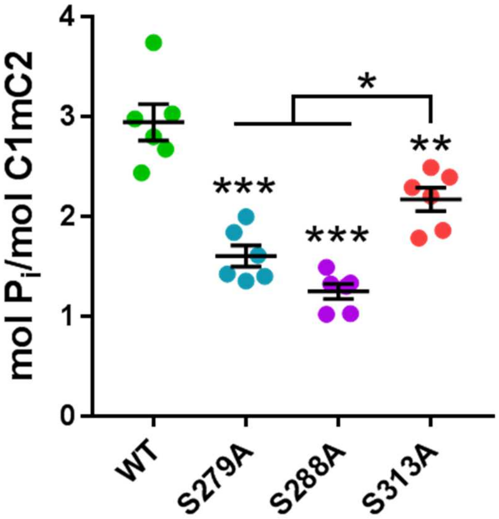

Fig. S2. Effects of serine-to-aspartate substitutions of PKA target sites in C1mC2 on phosphate incorporation by PKA. Phosphate incorporation was determined by PhostagTM-SDS-PAGE after incubation of 40 µmol/L C1mC2 constructs with 2000 U/ml PKA for 60 min. Statistical significance of differences between values was assessed with a one-way ANOVA followed by Tukey's post-hoc test:*p<0.05, **P<0.01, ***p<0.001.

a

C1mC2

```
        10         20         30         40         50         60         70         80
GSSMDDPIGL FLMRPQDGEV TVGGSIVFSA RVAGASLLKP PVVKWFKGKW VDLSSKVGQH LQLHDSYDRA SKVYLFELHI
        90        100        110        120        130        140        150        160
TDAQATSAGG YRCEVSTKDK FDSCNFNLTV HEAIGSGDLD LRSAFRRTSL AGTGRRTSDS HEDAGTLDFS SLLKKRDSFR
       170        180        190        200        210        220        230        240
RDSKLEAPAE EDVWEILRQA PPSEYERIAF QHGVTDLRGM LKRLKGMKHD EKKSTAFQKK LEPAYQVNKG HKIRLTVELA
       250        260        270        280        290        300
DPDAEVKWLK NGQEIQMSGS KYIFESIGAK RTLTISQCSL ADDAAYQCVV GGEKCSTELF VKE
```

b

RSK2

Mass (Da)	Peptide	Position	Best Expect Score	Phosphorylation Position	Localisation Probility (%)	Technique
2001.86	TSDSHEDAGTLDFSSLLK or TSDSHEDAGTLDFSSLLK	137-154	3.40E-05	T137 or S138	54 (S138) or 41.9 (T137)	MALDI-MS/MS
2157.97	RTSDSHEDAGTLDFSSLLK	136-154	1.20E-07	S138	90	MALDI-MS/MS

c

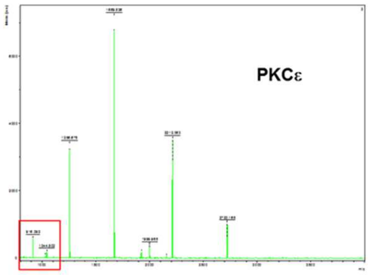

m/z	S/N	Quality Fac.	Res.	Intens.	Area
916.392	77.2	682	9455	551.78	102
1027.541	7	289	5788	48.93	17
1044.502	24.5	286	10729	167.69	33
1256.678	565.2	5732	11457	3379.61	893
1669.836	1569.5	14408	12260	7194.7	3008
1922.874	37	2101	15632	152.67	63
1999.968	76.6	4241	14266	291.79	138
2158.905	11.4	341	15161	39.7	19
2212.063	839.4	3850	13053	2870.34	1730
2723.168	223.8	1004	14487	588.08	422

User mass	DB mass	mass diff.	mod. diff.	Δmass (Da)	potential mod.	#MC	peptide	position
916.392	836.449	79.9435	79.9663	0.023	PHOS	2	RDSFRR	156-161
1044.5	964.544	79.9585	79.9663	0.008	PHOS	3	KRDSFRR	155-161

Fig. S3. Mass spectrometry analysis of RSK2 and PKCε phosphorylated C1mC2. (a) Rat C1mC2 primary sequence with phosphorylation sites highlighted in red. (b) Phosphorylation site identification of purified RSK2 phosphorylated C1mC2 by MALDI-MS/MS. (C) Phosphorylation site identification of purified and PKCε phosphorylated C1mC2 by ESI-MS/MS. The red box indicates unique masses corresponding to phosphorylated peptides shown in the bottom table. The numbering of phosphorylated positions corresponds to the amino acid position in C1mC2 shown in (a).

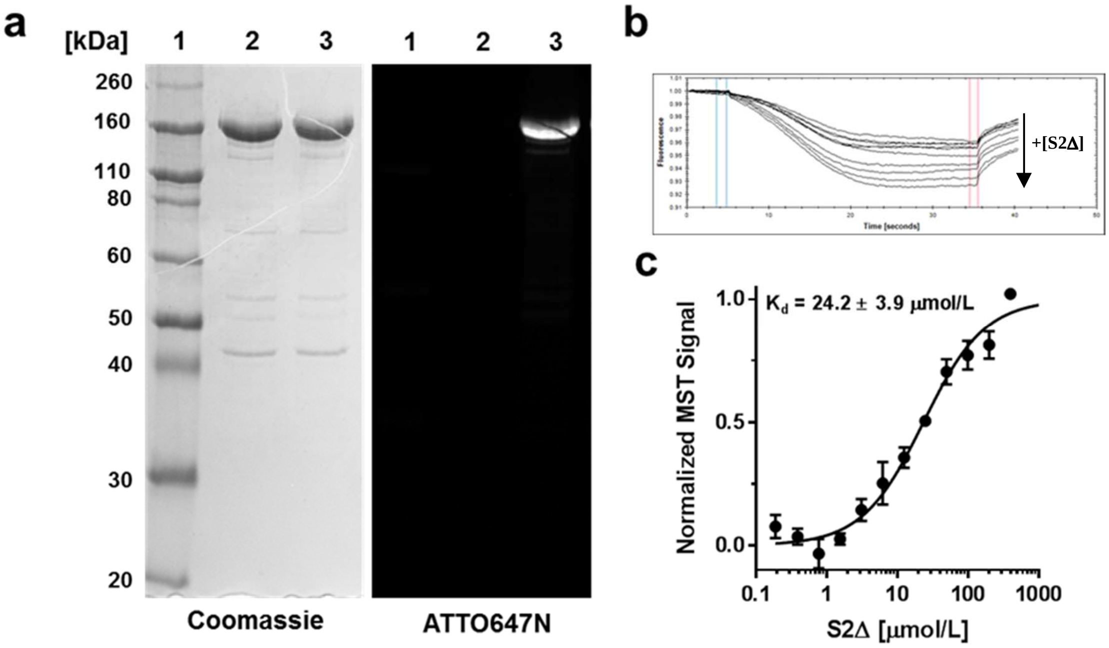

Fig. S4. Native cardiac myosin binding protein-C binding to myosin S2Δ. (a) SDS-PAGE of purified native rat cMyBP-C (lane 1-molecular weight marker; lane 2-purified cMyBP-C before labelling; lane 3-purified cMyBP-C after labelling with ATTO647N-maleimide). Bands were visualized by either coomassie staining (left) or ATTO647N fluorescence (right). (B) MST raw traces for myosin S2Δ titrated against native cMyBP-C-ATTO647N. Binding of myosin S2Δ to ATTO647N-labelled native cMyBP-C increases its thermophoretic mobility (indicated by arrow). (c) Normalized MST binding curve for native cMyBP-C binding to myosin S2Δ. The difference in thermophoretic mobility between the blue and red area shown in (B) for each myosin S2Δ concentration was fitted a single-binding-site isotherm and normalized. Means ± SEM, n=4.

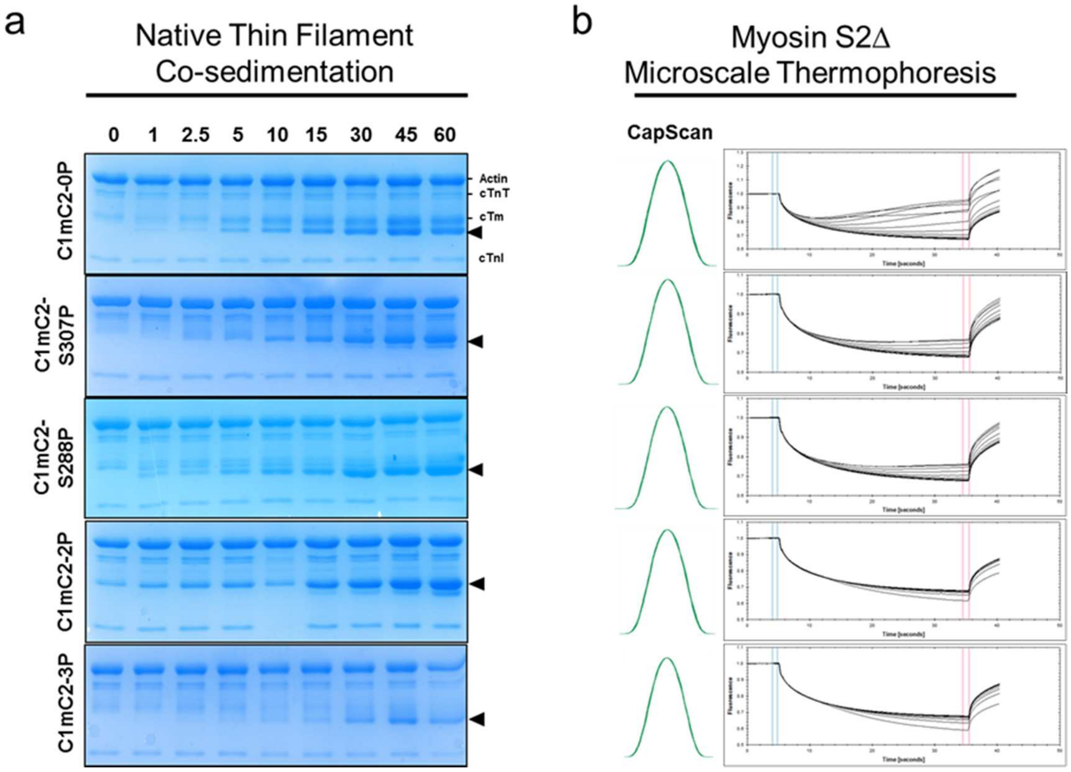

Fig. S5. C1mC2 binding to native thin filaments and myosin S2 Δ. (a) Representative SDS-PAGE analysis of co-sedimentation binding experiments between C1mC2 in its different phosphorylation states and isolated native thin filaments. Arrowheads indicate the position of C1mC2 constructs. (b) Representative MST capillary scans and raw traces for C1mC2 binding to myosin S2 Δ. CapScans on the left indicate no unspecific binding of proteins to the capillary walls. The original MST traces on the right show no aberrant behaviour, indicating no protein aggregation during the experiments.

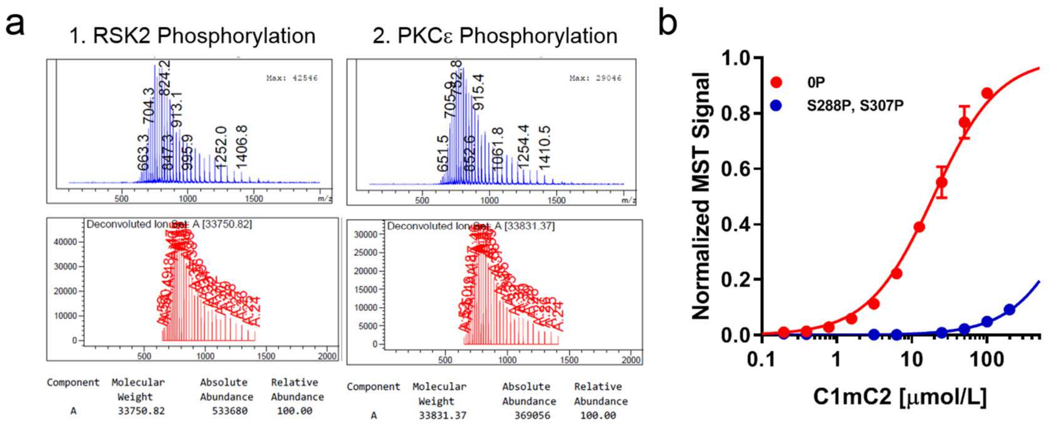

Fig. S6. Sequential phosphorylation of C1mC2 by RSK2 and PKCε. (a) ESI mass spectrometry analysis of purified C1mC2 after RSK2 phosphorylation (left) followed by PKCε phosphorylation (right). (b) Normalized MST binding curves for unphosphorylated C1mC2 (red) and C1mC2-S288P/S308P (blue) titrated against myosin S2Δ.

a

$$\mathrm{cMyBP\text{-}C\text{-}S2} \overset{K_{d,S2}}{\longleftrightarrow} \mathrm{S2}_{free} + \mathrm{cMyBP\text{-}C}_{free} + \mathrm{actin}_{free} \overset{K_{d,actin}}{\longleftrightarrow} \mathrm{cMyBP\text{-}C\text{-}actin}$$

b

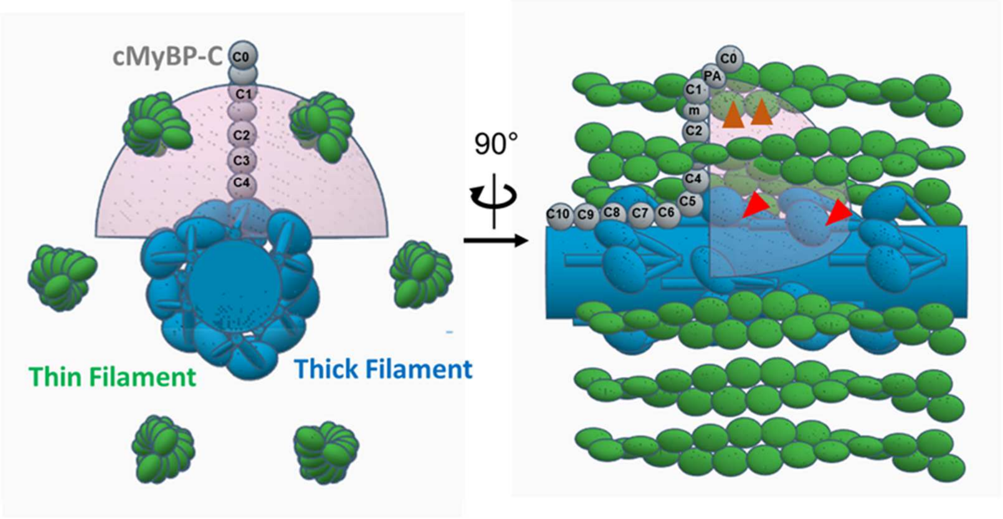

Fig. S7. Competition model for the interaction of cMyBP-C's N-terminal domains with the actin-containing thin and myosin-containing thick filaments. (a) Generalized scheme for the competitive interaction of cMyBP-C's N-terminal domains with either actin or myosin S2. Please note that a single cMyBP-C can either bind actin or myosin S2, therefore: [cMyBP-C]total=[cMyBP-C]free+[cMyBP-C-S2]+[cMyBP-C-actin]. (b) 3D-model of cMyBP-C and associated thick and thin filaments. Domains C0 through C10 are shown as grey spheres with a diameter of 4 nm and labelled accordingly. The thick filament backbone (14 nm diameter) and myosin head pairs are shown blue. Double stranded actin filaments are shown in green, with an actin monomer length of 5.5 nm and filament diameter of 10 nm. The pink shaded area indicates the estimated volume accessible to cMyBP-C's N-terminal domains including C1. Available actin and myosin S2 binding sites are indicated by brown and red arrowheads, respectively. For a more detailed description please see supplementary text.

Table S1. LC-MS analysis of phosphorylated and purified C1mC2 constructs. Δm denotes the mass differences to the unphosphorylated C1mC2.

	Calculated Mass [Da]	Measured Mass [Da]	Δm [Da]	# P
**C1mC2**	33671.05	33669.57	**-**	**0**
**+PKA**		33750.25	80.68	**1**
		33831.39	161.82	**2**
		33910.92	241.35	**3**
**+RSK2**		33749.83	80.26	**1**
**+PKCε**		33750.12	80.55	**1**
**+RSK2/ PKCε**		33831.37	161.80	**2**
**C1mC2-S288A**	33655.05	33655.49	-	**0**
**+PKA**		33735.18	79.69	**1**

Table S2. Steady-state equilibrium dissociation constants (K_d) and maximal binding capacity (B_{max}) for C1mC2 in its different phosphorylation states binding to myosin S2D and native thin filaments.

C1mC2	Myosin S2Δ	Native thin filaments			
		pCa 9		pCa 4.5	
	K_d [mmol/L]	K_d [mmol/L]	B_{max} [mol/mol]	K_d [mmol/L]	B_{max} [mol/mol]
0P	20.7 ± 3.5	23.0 ± 1.9	0.83 ± 0.1	16.9 ± 2.0	0.75 ± 0.09
S308P	33.9 ± 3.2*	20.3 ± 4.6	0.57 ± 0.13	24.3 ± 6.5	0.52 ± 0.09
S288P	30.6 ± 2.8*	25.3 ± 6.6	0.51 ± 0.07	15.6 ± 0.3	0.52 ± 0.07
S288P S279P	cd	13.0 ± 1.6	0.51 ± 0.02	16.3 ± 5.6	0.50 ± 0.08
S288P S279P S313P	cd	57.8 ± 5.1**	0.71 ± 0.07	62.6 ± 11.5**	1.1 ± 0.08

Means ± SEM (n=4-12). Statistical significance of differences from 0P was assessed by a one-way ANOVA followed by Tukey's post-hoc test:*$p<0.05$, **$p<0.01$. cd - Kd cannot be reliably determined

Supplemental Information References

1. Gilbert R, Cohen JA, Pardo S, Basu A, Fischman DA. Identification of the A-band localization domain of myosin binding proteins C and H (MyBP-C, MyBP-H) in skeletal muscle. *Journal of Cell Science* **112 (Pt 1)**, 69-79 (1999).
2. Al-Khayat HA, Kensler RW, Squire JM, Marston SB, Morris EP. Atomic model of the human cardiac muscle myosin filament. *Proceedings of the National Academy of Sciences of the United States of America* **110**, 318-323 (2013).
3. Previs MJ, *et al.* Phosphorylation and calcium antagonistically tune myosin-binding protein C's structure and function. *Proceedings of the National Academy of Sciences of the United States of America* **113**, 3239-3244 (2016).
4. Jeffries CM, Whitten AE, Harris SP, Trewhella J. Small-angle X-ray scattering reveals the N-terminal domain organization of cardiac myosin binding protein C. *Journal of Molecular Biology* **377**, 1186-1199 (2008).
5. Zoghbi ME, Woodhead JL, Moss RL, Craig R. Three-dimensional structure of vertebrate cardiac muscle myosin filaments. *Proceedings of the National Academy of Sciences of the United States of America* **105**, 2386-2390 (2008).
6. Kampourakis T, Ponnam S, Sun YB, Sevrieva I, Irving M. Structural and functional effects of myosin binding protein-C phosphorylation in heart muscle are not mimicked by serine-to-aspartate substitutions. *The Journal of Biological Chemistry*, (2018).
7. Kinoshita E, Kinoshita-Kikuta E, Takiyama K, Koike T. Phosphate-binding tag, a new tool to visualize phosphorylated proteins. *Molecular & Cellular Proteomics : MCP* **5**, 749-757 (2006).
8. Larsen MR, Thingholm TE, Jensen ON, Roepstorff P, Jorgensen TJ. Highly selective enrichment of phosphorylated peptides from peptide mixtures using titanium dioxide microcolumns. *Molecular & Cellular Proteomics : MCP* **4**, 873-886 (2005).
9. Savitski MM, *et al.* Confident phosphorylation site localization using the Mascot Delta Score. *Molecular & Cellular Proteomics : MCP* **10**, M110 003830 (2011).
10. Sun YB, Lou F, Irving M. Calcium- and myosin-dependent changes in troponin structure during activation of heart muscle. *The Journal of Physiology* **587**, 155-163 (2009).
11. Kampourakis T, Sun YB, Irving M. Orientation of the N- and C-terminal lobes of the Myosin regulatory light chain in cardiac muscle. *Biophysical Journal* **108**, 304-314 (2015).
12. Sevrieva I, Knowles AC, Kampourakis T, Sun YB. Regulatory domain of troponin moves dynamically during activation of cardiac muscle. *Journal of Molecular and Cellular Cardiology* **75**, 181-187 (2014).
13. Kampourakis T, Yan Z, Gautel M, Sun YB, Irving M. Myosin binding protein-C activates thin filaments and inhibits thick filaments in heart muscle cells. *Proceedings of the National Academy of Sciences of the United States of America* **111**, 18763-18768 (2014).
14. Kampourakis T, Sun YB, Irving M. Myosin light chain phosphorylation enhances contraction of heart muscle via structural changes in both thick and thin filaments. *Proceedings of the National Academy of Sciences of the United States of America*, (2016).
15. Corrie JE, *et al.* Dynamic measurement of myosin light-chain-domain tilt and twist in muscle contraction. *Nature* **400**, 425-430 (1999).
16. Brack AS, Brandmeier BD, Ferguson RE, Criddle S, Dale RE, Irving M. Bifunctional rhodamine probes of Myosin regulatory light chain orientation in relaxed skeletal muscle fibers. *Biophysical Journal* **86**, 2329-2341 (2004).
17. Dale RE, Hopkins SC, an der Heide UA, Marszalek T, Irving M, Goldman YE. Model-independent analysis of the orientation of fluorescent probes with restricted mobility in muscle fibers. *Biophysical Journal* **76**, 1606-1618 (1999).
